# Transcriptome analysis of the Chinese giant salamander (*Andrias davidianus*) using RNA-sequencing

**DOI:** 10.1016/j.gdata.2017.10.005

**Published:** 2017-10-28

**Authors:** Yong Huang, Jian Li Xiong, Xiao Chan Gao, Xi Hong Sun

**Affiliations:** College of Animal Science and Technology, Henan University of Science and Technology, Luoyang 471023, China

**Keywords:** *Andrias davidianus*, Transcriptome, Unigenes, Gene annotation

## Abstract

The Chinese giant salamander (*Andrias davidianus*) is an economically important animal on academic value. However, the genomic information of this species has been less studied. In our study, the transcripts of *A. davidianus* were obtained by RNA-seq to conduct a transcriptomic analysis. In total 132,912 unigenes were generated with an average length of 690 bp and N50 of 1263 bp by de novo assembly using Trinity software. Using a sequence similarity search against the nine public databases (CDD, KOG, NR, NT, PFAM, Swiss-prot, TrEMBL, GO and KEGG databases), a total of 24,049, 18,406, 36,711, 15,858, 20,500, 27,515, 36,705, 28,879 and 10,958 unigenes were annotated in databases, respectively. Of these, 6323 unigenes were annotated in all database and 39,672 unigenes were annotated in at least one database. Blasted with KEGG pathway, 10,958 unigenes were annotated, and it was divided into 343 categories according to different pathways. In addition, we also identified 29,790 SSRs. This study provided a valuable resource for understanding transcriptomic information of *A. davidianus* and laid a foundation for further research on functional gene cloning, genomics, genetic diversity analysis and molecular marker exploitation in *A. davidianus*.

## 1 Introduction

The Chinese giant salamander (*A. davidianus*) is the largest extant amphibian in the world [1], [2]. Now, it is classified as an endangered species by the International Union for Conservation of Nature and Nature Resources, and is the class II state major protection species in China. In the evolution history of vertebrate, *A. davidianus* occupies a seat at the phylogenetic and species evolution process which is representing a transitional form that links the aquatic animals to terrestrial organisms because it has existed for > 350 million years [3]. Therefore, this species has an important value in scientific research. In history, *A. davidianus* was widely distributed in central and southern China. However, in the past few decades, *A. davidianus* population has declined sharply due to deterioration of habitat, environmental pollution, climate change, infectious diseases, commercial trade and infrastructure development for human settlement [4], [5].

Owing to little genomic data was available previously for *A. davidianus*, it has hindered the understanding of the molecular mechanisms associated with growth, reproduction, immunization and sex determination of *A. davidianus*. In recent years, RNA sequencing technologies has been accepted as a powerful approach for large-scale transcriptome profiling for studying non-model species [6], [7], which has improved the efficiency and speed of gene discovery. Compared to the whole genome sequencing, RNA sequencing technologies provide a cost-effective approach to produce transcriptome sequences and molecule markers [8], [9], [10]. For example, a few of amphibians were undertaken a large-scale analysis of transcriptome sequenced by RNA sequencing technologies [11], [12]. Currently, transcriptome analysis reports of *A. davidianus* were only focused on skin, spleen, kidney, liver, intestines and gonad tissues [13], [14]. Therefore, further enriching the transcriptome analysis of *A. davidianus* has significant scientific value.

In this study, we are the first to characterize complete transcriptome of *A. davidianus* through the analysis of large-scale transcript sequences generated from a pooled mixed tissues including the spleen, liver, muscle, kidney, skin, testis, gut and heart by using the Illumina Hiseq 2500 high-throughput sequencing platform. These analyses identified a substantial number of unigenes which significantly improve our understanding on the genome prints of *A. davidianus*. Our results provide a global view of the transcriptome and pave the way for further functional characterization of *A. davidianus*.

## 2 Materials and methods

### 2.1 Ethics statement and sample collection

The healthy second generation of the farmed male *A. davidianus* (three years old) was obtained from Luoyang Huani Bio-Tech Co., Ltd. (Luoyang City, Henan, China). This study has also been reviewed and approved by the Ethics Committee of Henan University of Science and Technology according to the Regulations for the Administration of Affairs Concerning Experimental Animals (Ministry of Science and Technology, China; revised in June 2004). Subsequently, *A. davidianus* were anesthetized and sacrificed by decapitation. The organ samples including the spleen, liver, muscle, kidney, skin, testis, gut and heart were pooled and frozen at − 80 °C until RNA extraction.

### 2.2 RNA extraction and Illumina sequencing

Total RNA was extracted using TRIzol Reagent (Invitrogen, CA, USA) according to the manufacturers' directions, and then treated with RNase-free DNaseI. The extracted RNA content, integrity and purity were checked by a 2100 Bioanalyzer (Agilent Technologies). The pooled sample, 10 mg of total RNA was used for cDNA library construction following the protocol supplied with the Truseq™ RNA sample prep Kit. Briefly, Poly (A) mRNA was isolated using oligodT beads. All mRNA was broken into short fragments (200 nt) by adding fragmentation buffer. First-strand cDNA was generated using random primers and reverse transcriptase, then the second-strand cDNA was obtained with RNase H and DNA polymerase I. The cDNA fragments were purified using a QIAquick PCR extraction kit and washed with EB buffer for end reparation poly (A) addition. After that, the cDNA fragments were ligated to sequencing adapters. PCR amplification was then performed by selecting suitable fragments as templates. Finally, the cDNA library of *A. davidianus* was constructed and sequenced on the Illumina HiSeq 2500 platform (Sangon Biotech Co., Ltd., Shanghai, China). All raw data for *A. davidianus* obtained in this study were deposited in the National Center for Biotechnology Information (NCBI) Sequence Read Archive (SRA) with the accession numbers SRP099564.

### 2.3 Sequence data processing and de novo assembly

The quality of paired-end raw reads in fastq format was assessed using FastQC software (http://www.bioinformatics.babraham.ac.uk). Low-quality reads, such as adaptor sequences or with unknown nucleotides > 10%, were filtered. The clean reads were then combined to form longer fragments. Transcriptome de novo assembly was carried out using the short read assembly program Trinity with default settings to generate transcript. Finally, the redundancy in these transcripts was removed, and contigs were connected to get unique unigenes. All unigenes were predicted using ORF finder (http://www.ncbi.nlm.nih.gov/gorf/gorf.html).

### 2.4 Annotation of unigenes

Functional annotation of the unigenes was performed by search against the nine public databases. All unigenes were compared with sequences in Nr (NCBI non-redundant protein database), Nt (NCBI non-redundant nucleotide sequence database), KOG (EuKaryotic Orthologous Groups), CDD (Conserved Domains Database), PFAM (The Protein Families), GO (Gene Ontology), KEGG (Kyoto Encyclopedia of Genes and Genomes), Swiss-prot and TrEMBL with E-values ≤ 10^− 5^.

## 3 Results

### 3.1 Transcriptome and assembly characterization

**Fig. 1:**
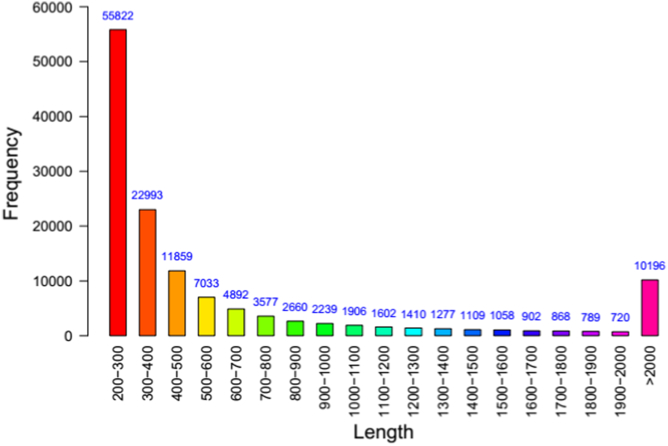
Assembled Unigenes length distribution of *A. davidianus* transcriptome.

**Fig. 2:**
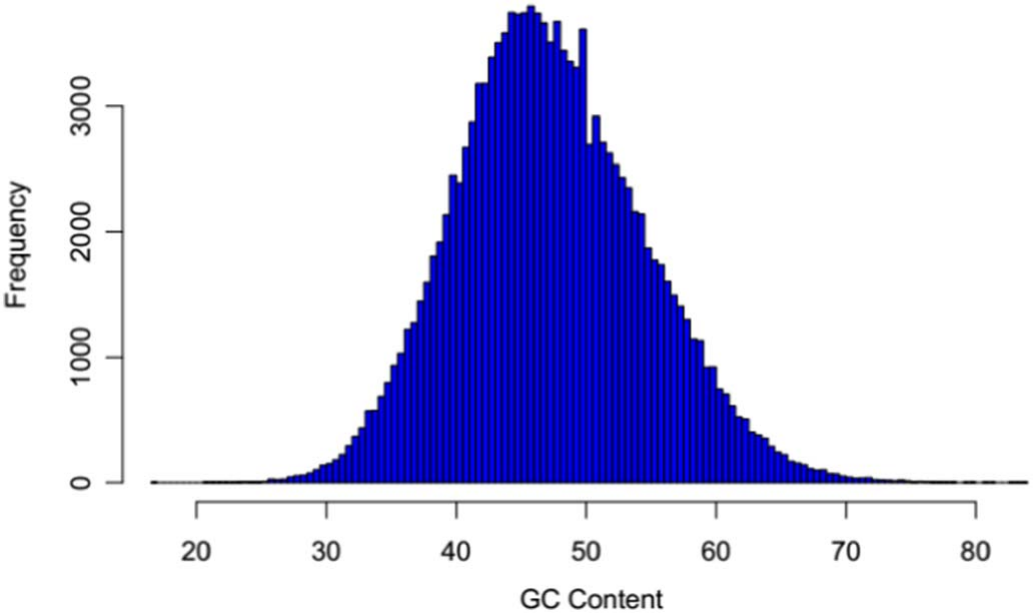
GC content distribution of unigenes.

The RIN value of total RNA form *A. davidianus* was 8.2 and the 28S/18S ratios also was 1.80. The cDNA library from *A. davidianus* was constructed and sequenced, which generated 102,659,984 raw reads containing 15,398,997,600 bp with an average length of 150 bp. After stringent quality assessment and data filtering, reads with Q20 bases (those with a base quality > 20) were selected as high quality reads for further analysis. Using the clean reads, Trinity produced 158,103 transcripts with an average length of 810 bp and N50 length of 1659 bp. The length of transcripts ranged from 201 to 16,067 bp. Finally, de novo assembly yielded 132,912 unigenes with an average length of 690 bp and N50 length of 1263 bp. Of these unigenes, 42,327 unigenes (31.85%) were > 500 bp and 21,855 unigenes (16.44%) were > 1000 bp (Table 1). As shown in Fig. 1, the sequence length of these unigenes ranged from 200 bp to > 2000 bp. The number of unigenes decreased with increasing length. In addition, the GC content is also one of the important characteristics of the genome base sequence, which can reflect the structure, function and evolutionary information of the gene. The heterogeneity of GC distribution may lead to the functional difference. The average content of GC of *A. davidianus* was 49.85%, and the unigenes with high GC content (> 80%) or too low (< 20%) did not found. The GC content met normal distribution (Fig. 2), indicating that the sequencing quality was perfect.

**Table 1:** Transcriptome assembly statistics in *A. davidianus*.

Category	Transcripts	Unigenes
Total length (bp)	128,175,999	91,713,308
Sequence no.	158,103	132,912
≥ 500 bp	59,715	42,327
≥ 1000 bp	34,075	21,855
N50	1659	1263
Max length (bp)	16,067	16,067
Min length (bp)	201	201
Average length (bp)	810	690

N50 of Transcripts or unigenes was calculated by ordering all sequences, then adding the lengths from longest to shortest until the summed length exceeded 50% of the total length of all sequence.

### 3.2 ORF prediction and annotation of unigenes sequences

**Fig. 3:**
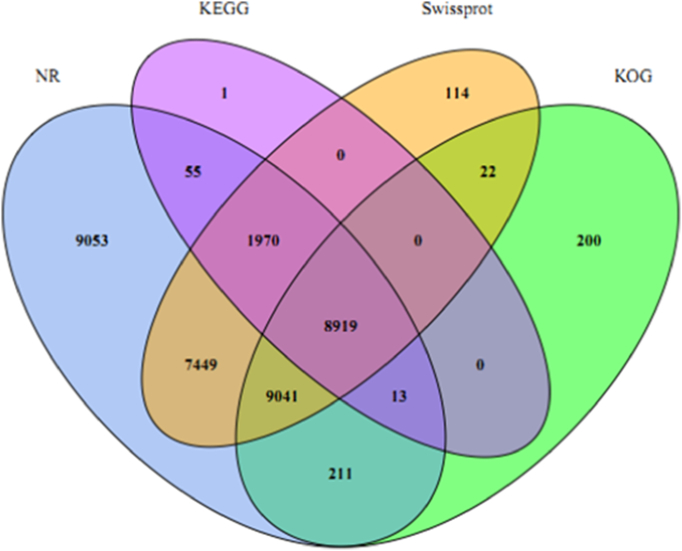
Venn diagram shows commonality and difference of annotation based on NR, KEGG, Swiss-Prot and KOG.

The ORF sequence was predicted by using ORF prediction software, and 132,416 were predicted to be encoded amino acids, accounting for 99.63% of all unigenes. The remaining 496 unigenes contained no ORFs, indicating they may either be non-coding sequences coming from untranslated regions (UTR) or de novo unigenes that contain < 150 bp of the start or end of an ORF. Using the BLASTx tool, the unigenes were aligned with sequences recorded in the major databases including CDD, KOG, NR, NT, Pfam, Swisse-Prot, TrEMBL, GO and KEGG. A Venn diagram illustrates the distribution of unigenes annotated to four databases (Fig. 3). Among 132,912 unigenes, a total of 24,049 showed significant matches to CDD, 18,406 to KOG, 36,711 to NR, 15,858 to NT, 20,500 to Pfam, 27,515 to Swiss-Prot, 36,705 to TrEMBL, 28,879 to GO and 10,958 to KEGG respectively. Altogether, 6323 (4.76%) unigenes exhibited a significant match with nine major public databases, and 39,672 unigenes showed significant match, at least one hit to these databases (Table 2).

**Table 2:** Unigenes functional annotation by various databases.

Databases	Number of unigenes	Percentage (%)
CDD	24,049	18.09
KOG	18,406	13.85
NR	36,711	27.62
NT	15,858	11.93
Pfam	20,500	15.42
Swiss-prot	27,515	20.7
TrEMBL	36,705	27.62
GO	28,879	21.73
KEGG	10,958	8.24
Annotated in at least one database	39,672	29.85
Annotated in all database	6323	4.76
All unigenes	132,912	100

### 3.3 Functional classification by GO, KOG and KEGG

**Fig. 4:**
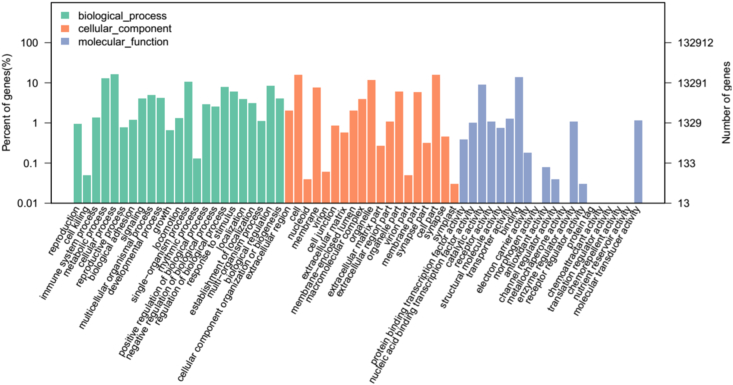
GO functional annotation of unigenes.

GO provides an international standardized gene functional classification system of each assembled unigenes by blasting with the Nr database. In this study, a total of 41,553 unigenes were categorized into 62 subcategories under three main ontologies: molecular function, cellular component, and biological process. For biological process, 21,763 (16.37%) were in the cellular process category, 17,266 (12.99%) were in the metabolic process category and 13,952 (10.50%) were in the single-organism process category. For cellular component, (20,759, 15.62%) cell part and cell represented the majority of this category, respectively. Meanwhile, for molecular function, 18,579 (13.98%) binding and 12,120 (9.12%) catalytic activity were highly represented and assigned to this category, whereas only a few genes were assigned to the metallochaperone activity (6), morphogenesis activity (5) and protein tag (4) (Fig. 4). All of these results indicated that a large fraction of unigenes function differentially and interdependently in *A. davidianus* organism.

**Fig. 5:**
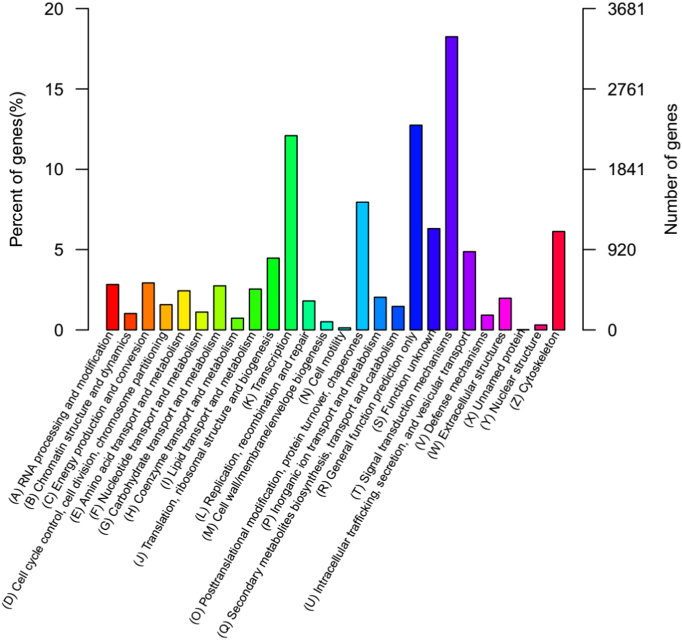
KOG annotation of unigenes.

KOG is a database that classifies orthologous gene products. We mapped all the unigenes to the KOG database to predict the possible functions and statistics and to elucidate gene function distribution characteristics of species at the macro level. In total, 18,406 unigenes were assigned to the KOG database and classified into 26 KOG categories (Fig. 5). Of the 26 categories, the cluster for signal transduction mechanism represented the largest group (3357, 18.24%), followed by general functional prediction (2345, 12.74%), transcript (2226, 12.09%), cytoskeleton (1129, 6.13%), cell endocrine and vesicular transport (897, 4.87%) and cell motility (24, 0.13%). Only a few unigenes were assigned to unknown protein (4, 0.02%). These results were slightly different than those obtained from previously study in *A. davidianus* [13].

**Fig. 6:**
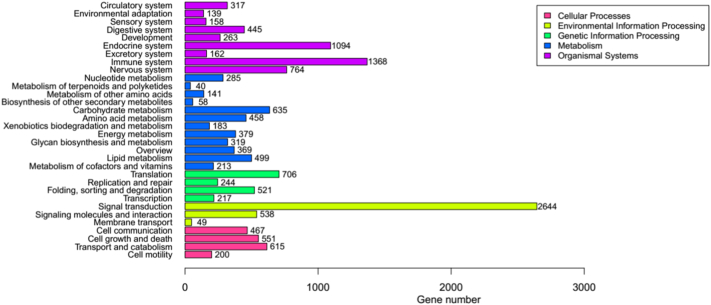
KEGG annotation of unigenes.

KEGG is widely used as a reference database of pathway networks for integration and interpretation of large-scale datasets generated by RNA sequencing technology. In order to understand the function of sequenced genes in *A. davidianus*, all the unigenes were compared with KEGG using BLASTx, and the corresponding pathways were elucidated. A total of 10,958 (8.24%) unigenes were annotated to 32 categories in the KEGG database from 5 main groups (Cellular Processes, Environmental Information Processing, Genetic Information Processing, Metabolism and Organismal Systems) and located to 343 known KEGG pathways (Fig. 6). The largest cluster was ‘signal transduction’ (2644, 17.58%), followed by ‘immune system’ (1368, 9.10%), and ‘endocrine system’ (1094, 7.27%). In terms of signal transduction, the PI3K-Akt, MAPK, Rap1, Ras, cGMP-PKG, Calcium, FoxO, Hippo, Wnt, TNF, and NF-kappa B signaling pathways were found from transcriptomes of *A. davidianus*, indicating that a large number of signal generations occur during the development of the *A. davidianus*.

### 3.4 Identification of SSR markers

Due to the stability and extensive distribution of microsatellite marker (SSR) along genomes and transcriptomes, SSRs are still widely used in genetics and biology researches. In the present study, using the MISA Perl script, a total of 21,470 unigenes containing 29,790 SSRs were identified from 132,913 unigenes, of which 5644 sequences contained more than one SSR (Table 3). The result of amount of SSRs can be similar to the previous research of *A. davidianus* (31,982 SSRs were detected in 24,131 unigenes) [13]. The most abundant repeat type was single-nucleotide, A/T (25,100, 84.25%), di-nucleotide GT/AC (3276, 11.0%), followed by the tri-nucleotide, TGC/GCA (1237, 4.15%) and tetra-nucleotide GCTA (168, 0.56%). Since SSRs were ubiquitous in transcriptomes, and were typically locus-specific, co-dominant, multiallelic, and highly polymorphic, they have been developed as powerful molecular markers for comparative genetic mapping and genotyping. Therefore, the unigenes obtained in this study provided a good resource for SSRs application in marker-assisted breeding from *A. davidianus*.

**Table 3:** General statistics of SSR identified transcriptome.

Item	Number
Total number of identified SSR	29,790
Number of SSR containing sequences	21,470
Number of sequences containing > 1 SSR	5644
Number of SSRs present in compound formation	1923
Mononucleotide	25,100
Dinucleotide	3276
Trinucleotide	1237
Tetranucleotide	168
Pentanucleotide	8
Hexanucleotide	1

## 4 Discussion

RNA-Seq technique enabled deep transcriptome analysis of many kinds of organisms, which has many advantages, such as low cost, capacity of large amount of information, high sensitivity and easy detection of the existence of low-expression genes. It plays an important role in the revelation of the complexity of transcriptome, the identification of new genes and non-coding RNA, as well as the exploitation of related-molecular marker. Currently, a few studies in transcriptomes of *A. davidianus* were reported. Li et al. identify 57,654 and 64,807 unigenes from the skin and spleen tissues of *A. davidianus* using this technology, respectively [14], [15]. Jiang et al. also performed transcriptome analysis from the spleen tissue of *A. davidianus* and a total of 72,072 unigenes were obtained [16]. Fan et al. also conducted transcriptome analysis from the virus-infected and healthy spleen tissue of *A. davidianus* and 123,440 unigenes were obtained [17]. Similarity, Qi et al. also obtained 150,172 unigenes from the liver tissue of *A. davidianus* by using RNA-seq technology [18]. These studies provided an abundance of expressed sequences and identified many immune-related genes from transcriptome sequencing in *A. davidianus*, which will be valuable for further functional genomics research in *A. davidianus*. In our present study, transcriptomes from *A. davidianus* were comprehensively sequenced and analyzed. A total of 15,398,997,600 bp were generated, and were finally assembled to 132,912 unigenes with an average length of 690 bp. The average length of the transcripts was longer than that of previous studies using other de novo assembly methods [19], [20], [21]. It is noteworthy that 21,855 unigenes (16.44%) had lengths longer than 1000 bp, which is more than that of other organisms using Trinity for assembly [22], [23]. These numerous sequences can provide a sufficient transcriptome sequence resource for discovering novel genes in *A. davidianus*. These results showed that Trinity is a powerful and efficient tool for de novo assembly for organisms without reference genomes.

In our study, only 6323 unigenes were annotated in the 9 public databases. There maybe is a lack of available genomics information of *A. davidianus* in databases. Compared with other species, such as *Zebrafish*, *Silkworm*, *Fruit fly*, *Xenopus laevis* and so on, transcriptome study on *A. davidianus* is lags far behind. In the unigenes annotated in the NR database, the 18.87% of the unigenes matched proteins from *Vitis vinifera*. This result might be related to the evolutionary relationship, because it belongs to the reptile. However, majority (31.31%) unigenes were not matched in any species. These results indicated that the gene information of *A. davidianus* which exists in the database is very limited, making it difficult for transcriptomes annotation of *A. davidianus*. With annotations in the GO database, the large numbers of genes related to cell process, metabolic process, cell, cell assembly, binding and catalytic activity. Results implied that these transcripts may be genes which control specific cell proliferation and differentiation in *A. davidianus*, and therefore, will be useful for gene functional study. In the KOG database, a large number of unigenes were assigned to a great diversity of KOG classifications, and 13.85% of the unigenes were annotated indicating that our sequencing data represented a diverse range of transcripts. Among them, most unigenes mapped to the signal transduction mechanism. Results indicated that the transmission of these genes information were very active in the physiological activities of *A. davidianus*. KEGG metabolic pathway analysis showed that signal transduction genes constituted the largest second-tier hierarchical category of KEGG assignments. Of them, 1388 unigenes involved in the immune system, which are related to resistance to diseases and associated with antiviral immune factors, such as pattern recognition receptors, T cell activation antigen molecules, inflammatory cytokines and receptors, complement components, B cells, interferon and interferon-stimulated genes. Results were consistent with the previous studies [13]. These results also suggest that amphibians may have a large number of immune-related genes than fishes or mammals, which may contribute to better understanding the evolution of adaptive immunity against pathogens. Due to specific environment comparing to other aquatic organisms or amphibians, *A. davidianus* may develop its immune system as early as possible in order to defend against external pathogenic microbes or predators. Pathway-based analysis in this study helps to further elucidate the biological functions and interactions of genes in *A. davidianus*.

Unigenes were searched for SSRs markers using the MISA software and 29,790 SSRs were identified. The most abundant mononucleotide repeat type was A/T (25,100, 84.2%). Similar distribution of motif types and annotation rates were obtained in previous research from animals [15], [24], [25]. Given that the mononucleotide repeats may not be accurate as a result of sequencing errors and assembly mistakes, 25,100 SSRs that exclude mononucleotide repeats were detected, indicating the highly efficient discovery. So far, there is no publically available SSRs in *A. davidianus*, SSRs identified in this study may be useful for the development of large sets of markers which would facilitate linkage mapping studies, population genetics and functional genomics research on *A. davidianus* in the future.

## 5 Conclusion

In summary, we report the elucidation of the transcriptome of *A. davidianus*. After de novo assembly and sequence annotation, we obtained 132,912 unigenes. Of these unigenes, 28, 879 were annotated in GO database. The biological pathways involving some of these unigenes were also identified. Pathway-based analysis helps to further elucidate the biological functions and interactions of genes of *A. davidianus*. In addition, 29,790 SSRs were identified. Our data significantly enhance the molecular resources available for future study and provide insights into the genetic background in *A. davidianus*.

## Conflicts of interest

The authors declare no conflict of interest.
